# Dichlorido(dimethyl­formamide-κ*O*)[1,4,7-tris­(2-cyano­ethyl)-1,4,7-triaza­cyclo­nonane-κ^3^ *N* ^1^,*N* ^4^,*N* ^7^]cobalt(II)

**DOI:** 10.1107/S160053680802179X

**Published:** 2008-07-19

**Authors:** Zhong Zhang, Zhi-Rong Geng, Qun Zhao, Zhi-Lin Wang

**Affiliations:** a Coordination Chemistry Institute, State Key Laboratory of Coordination Chemistry, Nanjing University, Nanjing 210093, People’s Republic of China

## Abstract

The title compound, [CoCl_2_(C_15_H_24_N_6_)(C_3_H_7_NO)], crystallizes as a monomeric complex. The coordination environment around the Co^II^ center could be described as a distorted octa­hedron consisting of three N donors from the facially coordinating triaza macrocyclic ligand, one O donor from dimethyl­formamide and two Cl^−^ ions. Neutral complex mol­ecules are associated *via* inter­molecular C—H⋯Cl hydrogen bonds to form two-dimensional layers. C—H⋯O hydrogen bonds are also present.

## Related literature

For related literature, see: Scarpellini *et al.* (2005 ▶); Tei *et al.* (1998 ▶, 2003 ▶).
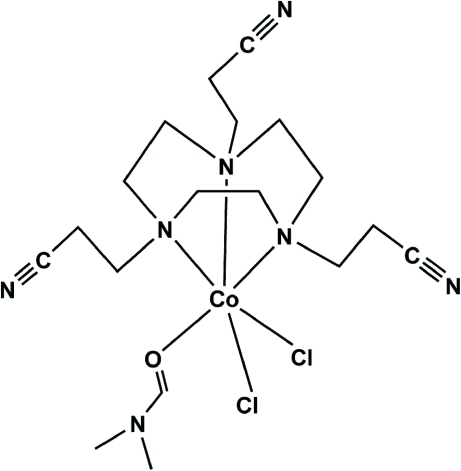


## Experimental

### 

#### Crystal data

[CoCl_2_(C_15_H_24_N_6_)(C_3_H_7_NO)]
*M* *_r_* = 491.33
Monoclinic, 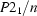

*a* = 9.787 (2) Å
*b* = 19.710 (5) Å
*c* = 12.370 (3) Å
β = 97.936 (4)°
*V* = 2363.5 (10) Å^3^
*Z* = 4
Mo *K*α radiation
μ = 0.97 mm^−1^
*T* = 298 (2) K
0.32 × 0.26 × 0.24 mm

#### Data collection

Bruker SMART APEX CCD area-detector diffractometer
Absorption correction: multi-scan (*SADABS*; Bruker, 2000 ▶) *T*_min_ = 0.746, *T*_max_ = 0.800
12542 measured reflections
4630 independent reflections
3320 reflections with *I* > 2σ(*I*)
*R*_int_ = 0.052

#### Refinement

*R*[*F* ^2^ > 2σ(*F* ^2^)] = 0.067
*wR*(*F* ^2^) = 0.154
*S* = 1.07
4630 reflections
264 parameters
H-atom parameters constrained
Δρ_max_ = 0.90 e Å^−3^
Δρ_min_ = −0.78 e Å^−3^

### 

Data collection: *SMART* (Bruker, 2000 ▶); cell refinement: *SAINT* (Bruker, 2000 ▶); data reduction: *SAINT*; program(s) used to solve structure: *SHELXTL* (Sheldrick, 2008 ▶); program(s) used to refine structure: *SHELXTL*; molecular graphics: *SHELXTL* and *ORTEP-3* (Farrugia, 1997 ▶); software used to prepare material for publication: *SHELXTL*.

## Supplementary Material

Crystal structure: contains datablocks I, global. DOI: 10.1107/S160053680802179X/ww2123sup1.cif

Structure factors: contains datablocks I. DOI: 10.1107/S160053680802179X/ww2123Isup2.hkl

Additional supplementary materials:  crystallographic information; 3D view; checkCIF report

## Figures and Tables

**Table:** 

Cl1—Co1	2.4382 (14)
Cl2—Co1	2.4096 (13)
Co1—O1	2.114 (3)
Co1—N2	2.194 (4)
Co1—N3	2.200 (4)
Co1—N1	2.232 (4)

**Table:** 

O1—Co1—N2	87.78 (12)
O1—Co1—N3	168.01 (13)
N2—Co1—N3	80.93 (13)
O1—Co1—N1	93.00 (13)
N2—Co1—N1	81.07 (14)
N3—Co1—N1	81.30 (13)
O1—Co1—Cl2	90.45 (9)
N2—Co1—Cl2	173.80 (10)
N3—Co1—Cl2	100.34 (10)
N1—Co1—Cl2	93.09 (10)
O1—Co1—Cl1	91.12 (9)
N2—Co1—Cl1	93.38 (10)
N3—Co1—Cl1	93.58 (10)
N1—Co1—Cl1	172.94 (10)
Cl2—Co1—Cl1	92.60 (4)

**Table 2:** Hydrogen-bond geometry (Å, °)

*D*—H⋯*A*	*D*—H	H⋯*A*	*D*⋯*A*	*D*—H⋯*A*
C1—H1*A*⋯Cl2^i^	0.97	2.75	3.658 (4)	157
C2—H2*A*⋯O1	0.97	2.58	3.120 (5)	116
C3—H3*A*⋯Cl2^i^	0.97	2.81	3.774 (4)	170
C7—H7*A*⋯Cl2	0.97	2.65	3.419 (4)	136
C10—H10*A*⋯O1	0.97	2.47	3.149 (6)	127
C10—H10*B*⋯Cl1	0.97	2.73	3.218 (4)	111
C11—H11*A*⋯Cl1^i^	0.97	2.62	3.525 (5)	156
C11—H11*B*⋯Cl2^ii^	0.97	2.65	3.502 (5)	147
C13—H13*A*⋯Cl1	0.97	2.72	3.460 (5)	133
C16—H16⋯Cl2	0.93	2.80	3.325 (5)	117
C17—H17*A*⋯O1	0.96	2.34	2.741 (7)	105

Symmetry codes: (i) 
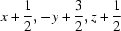
; (ii) 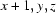.

**Fig. 1.**
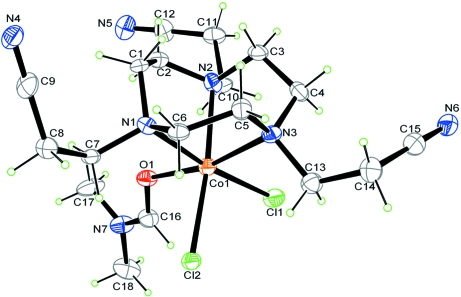
The molecular structure of the title compound, showing displacement ellipsoids at the 30% probability level (arbitrary sphere for H atoms).

**Fig. 2.**
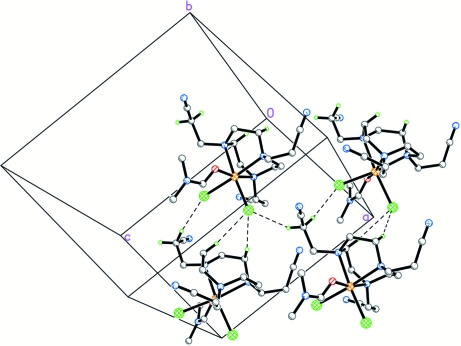
Partial packing diagram of the title compound, showing the two-dimensional network formed through intermolecular C—H···Cl hydrogen bonds (dashed lines). For clarity, H atoms not involved in hydrogen bonding have been omitted.

## supplementary crystallographic information

### Comment

Structural investigations on metal complexes with nitrile pendant arm derivatives of 1,4,7-triazacyclononane ([9]aneN_3_) reveal that these triazamacrocyclic ligands can either be used as building-blocks to assemble multi-dimensional polymeric networks (Tei *et al.*, 1998) or only act as tridentate ligands in the formation of mononuclear complexes with the pendant nitrile groups not involved in metal coordination (Tei *et al.*, 2003). In this paper, we report the crystal structure of the title compound, (I), which is a monomeric Co^II^ complex of a [9]aneN_3_ derivative containing three pendant 2-cyanoethyl arms.

The molecular structure of (I) (Fig. 1) shows the Co^II^ ion is six-coordinated with three tertiary N donors from the nitrile-functionalized [9]aneN_3_, one O donor from dimethylformamide ligand and two Cl^-^ ions completing an octahedral geometry. All bond distances and angles around the octahedral Co^II^ ion (Table 1) are generally within the normal ranges (Scarpellini *et al.*, 2005). Three pendant 2-cyanoethyl arms attached to the triazamacrocycle adopt different conformations relative to the macrocycle framework and none of them participates in the coordination to the Co^II^ ion.

The crystal packing of (I) is dominated by intermolecular C—H···Cl hydrogen bonds (Table 2), which link the complex molecules to form two-dimensional hydrogen-bonded layers parallel to (010) plane (Fig. 2).

### Experimental

The triazamacrocyclic ligand 1,4,7-tris(2-cyanoethyl)-1,4,7-triazacyclononane was prepared following a literature procedure (Tei *et al.*, 1998). A mixture of the triazamacrocyclic ligand (29 mg, 0.1 mmol) and CoCl_2_.6H_2_O (24 mg, 0.1 mmol) in  MeOH (10 ml) was stirred under reflux for 2 h. The precipitated pink solid was filtered off and subsequently redissolved in dimethylformamide. Purple single crystals of (I) suitable for X-ray diffraction analysis were obtained by slow diffusion of diethyl ether into the dimethylformamide solution. (yield 23 mg, 46.8%) Elemental analysis found: C 44.13; H 6.41; N 19.81%; calculated for C_18_H_31_Cl_2_CoN_7_O: C 44.00; H 6.36; N 19.96%.

### Refinement

All H atoms were placed in calculated positions and treated in the subsequent refinement as riding atoms, with C—H distances in the range 0.96 - 0.97 Å and *U*_iso_(H) = 1.2 *U*_eq_(C) or 1.5 *U*_eq_(methyl C).

### Crystal data

**Table:**

[CoCl_2_(C_15_H_24_N_6_)(C_3_H_7_NO)]	*F*_000_ = 1028
*M**_r_* = 491.33	*D*_x_ = 1.381 Mg m^−^^3^
Monoclinic, *P*2_1_/*n*	Mo *K*α radiation λ = 0.71073 Å
Hall symbol: -P 2yn	Cell parameters from 915 reflections
*a* = 9.787 (2) Å	θ = 3.2–26.3º
*b* = 19.710 (5) Å	µ = 0.98 mm^−^^1^
*c* = 12.370 (3) Å	*T* = 298 (2) K
β = 97.936 (4)º	Block, purple
*V* = 2363.5 (10) Å^3^	0.32 × 0.26 × 0.24 mm
*Z* = 4	

### Data collection

**Table:**

Bruker SMART APEX CCD area-detector diffractometer	4630 independent reflections
Radiation source: sealed tube	3320 reflections with *I* > 2σ(*I*)
Monochromator: graphite	*R*_int_ = 0.052
*T* = 298(2) K	θ_max_ = 26.0º
ω and φ scans	θ_min_ = 2.0º
Absorption correction: multi-scan(SADABS; Bruker, 2000)	*h* = −11→12
*T*_min_ = 0.746, *T*_max_ = 0.800	*k* = −22→24
12542 measured reflections	*l* = −14→15

### Refinement

**Table:**

Refinement on *F*^2^	Secondary atom site location: difference Fourier map
Least-squares matrix: full	Hydrogen site location: inferred from neighbouring sites
*R*[*F*^2^ > 2σ(*F*^2^)] = 0.067	H-atom parameters constrained
*wR*(*F*^2^) = 0.154	*w* = 1/[σ^2^(*F*_o_^2^) + (0.0683*P*)^2^ + 1.5989*P*] where *P* = (*F*_o_^2^ + 2*F*_c_^2^)/3
*S* = 1.07	(Δ/σ)_max_ < 0.001
4630 reflections	Δρ_max_ = 0.90 e Å^−^^3^
264 parameters	Δρ_min_ = −0.78 e Å^−^^3^
Primary atom site location: structure-invariant direct methods	Extinction correction: none

### Special details

**Table:**

Geometry. All e.s.d.'s (except the e.s.d. in the dihedral angle between two l.s. planes) are estimated using the full covariance matrix. The cell e.s.d.'s are taken into account individually in the estimation of e.s.d.'s in distances, angles and torsion angles; correlations between e.s.d.'s in cell parameters are only used when they are defined by crystal symmetry. An approximate (isotropic) treatment of cell e.s.d.'s is used for estimating e.s.d.'s involving l.s. planes.
Refinement. Refinement of *F*^2^ against ALL reflections. The weighted *R*-factor *wR* and goodness of fit *S* are based on *F*^2^, conventional *R*-factors *R* are based on *F*, with *F* set to zero for negative *F*^2^. The threshold expression of *F*^2^ > σ(*F*^2^) is used only for calculating *R*-factors(gt) *etc*. and is not relevant to the choice of reflections for refinement. *R*-factors based on *F*^2^ are statistically about twice as large as those based on *F*, and *R*- factors based on ALL data will be even larger.

### Fractional atomic coordinates and isotropic or equivalent isotropic displacement parameters (Å^2^)

**Table:**

	*x*	*y*	*z*	*U*_iso_*/*U*_eq_	
C1	0.2270 (4)	0.8211 (2)	0.3872 (3)	0.0324 (9)	
H1A	0.2422	0.7803	0.4311	0.039*	
H1B	0.2274	0.8595	0.4364	0.039*	
C2	0.3432 (4)	0.8292 (2)	0.3190 (4)	0.0361 (10)	
H2A	0.3394	0.8744	0.2878	0.043*	
H2B	0.4308	0.8245	0.3658	0.043*	
C3	0.3572 (5)	0.7079 (2)	0.2715 (3)	0.0379 (10)	
H3A	0.3539	0.7075	0.3495	0.046*	
H3B	0.4479	0.6923	0.2594	0.046*	
C4	0.2498 (5)	0.6602 (2)	0.2164 (4)	0.0389 (10)	
H4A	0.2658	0.6531	0.1415	0.047*	
H4B	0.2586	0.6167	0.2533	0.047*	
C5	0.0698 (5)	0.6922 (2)	0.3276 (4)	0.0373 (10)	
H5A	0.1510	0.6860	0.3812	0.045*	
H5B	0.0049	0.6564	0.3381	0.045*	
C6	0.0060 (4)	0.7591 (2)	0.3466 (3)	0.0303 (9)	
H6A	−0.0840	0.7617	0.3028	0.036*	
H6B	−0.0072	0.7624	0.4227	0.036*	
C7	0.0145 (4)	0.8814 (2)	0.3205 (3)	0.0304 (9)	
H7A	−0.0535	0.8830	0.2556	0.037*	
H7B	0.0790	0.9182	0.3148	0.037*	
C8	−0.0598 (5)	0.8954 (3)	0.4193 (3)	0.0381 (10)	
H8A	−0.1046	0.9393	0.4088	0.046*	
H8B	−0.1318	0.8617	0.4201	0.046*	
C9	0.0240 (6)	0.8954 (3)	0.5263 (4)	0.0466 (12)	
C10	0.4320 (4)	0.7942 (3)	0.1517 (3)	0.0385 (10)	
H10A	0.3992	0.8346	0.1115	0.046*	
H10B	0.4295	0.7572	0.0997	0.046*	
C11	0.5839 (5)	0.8060 (3)	0.2025 (4)	0.0446 (12)	
H11A	0.6046	0.7777	0.2669	0.054*	
H11B	0.6442	0.7920	0.1505	0.054*	
C12	0.6122 (6)	0.8753 (3)	0.2321 (5)	0.0509 (13)	
C13	0.0065 (5)	0.6472 (2)	0.1419 (4)	0.0413 (11)	
H13A	0.0278	0.6533	0.0683	0.050*	
H13B	−0.0832	0.6675	0.1447	0.050*	
C14	−0.0065 (6)	0.5711 (2)	0.1614 (5)	0.0510 (13)	
H14A	−0.0061	0.5636	0.2390	0.061*	
H14B	−0.0950	0.5559	0.1244	0.061*	
C15	0.0989 (6)	0.5305 (3)	0.1257 (5)	0.0522 (13)	
C16	0.1312 (5)	0.9111 (2)	0.0171 (4)	0.0413 (11)	
H16	0.0671	0.8850	−0.0274	0.050*	
C17	0.2703 (6)	1.0089 (3)	0.0374 (5)	0.0666 (17)	
H17A	0.2868	0.9934	0.1116	0.100*	
H17B	0.2374	1.0548	0.0355	0.100*	
H17C	0.3546	1.0068	0.0060	0.100*	
C18	0.1187 (6)	0.9877 (3)	−0.1311 (5)	0.0618 (16)	
H18A	0.0484	0.9566	−0.1617	0.093*	
H18B	0.1924	0.9880	−0.1749	0.093*	
H18C	0.0802	1.0325	−0.1296	0.093*	
Cl1	0.17722 (12)	0.74778 (6)	−0.02560 (9)	0.0444 (3)	
Cl2	−0.11597 (11)	0.80786 (5)	0.07777 (8)	0.0339 (2)	
Co1	0.12259 (6)	0.78880 (3)	0.14939 (5)	0.03397 (19)	
N1	0.0910 (4)	0.81680 (18)	0.3190 (3)	0.0329 (8)	
N2	0.3359 (4)	0.77830 (18)	0.2297 (3)	0.0371 (9)	
N3	0.1097 (4)	0.68609 (18)	0.2168 (3)	0.0319 (8)	
N4	0.0843 (4)	0.8938 (2)	0.6097 (3)	0.0441 (10)	
N5	0.6313 (5)	0.9298 (2)	0.2543 (4)	0.0551 (12)	
N6	0.1858 (5)	0.4987 (2)	0.0935 (4)	0.0492 (10)	
N7	0.1708 (4)	0.9672 (2)	−0.0227 (3)	0.0470 (10)	
O1	0.1717 (3)	0.88941 (16)	0.1096 (2)	0.0372 (7)	

### Atomic displacement parameters (Å^2^)

**Table:**

	*U*^11^	*U*^22^	*U*^33^	*U*^12^	*U*^13^	*U*^23^
C1	0.030 (2)	0.039 (2)	0.025 (2)	0.0038 (18)	−0.0088 (16)	−0.0029 (17)
C2	0.029 (2)	0.042 (2)	0.036 (2)	−0.0020 (18)	−0.0027 (17)	0.0042 (19)
C3	0.039 (2)	0.049 (3)	0.024 (2)	0.014 (2)	−0.0005 (18)	−0.0011 (19)
C4	0.046 (3)	0.035 (2)	0.036 (2)	0.013 (2)	0.006 (2)	0.0042 (19)
C5	0.046 (3)	0.035 (2)	0.032 (2)	0.001 (2)	0.0073 (19)	0.0015 (19)
C6	0.025 (2)	0.041 (2)	0.0255 (19)	0.0005 (18)	0.0034 (15)	0.0055 (17)
C7	0.025 (2)	0.041 (2)	0.024 (2)	0.0045 (17)	−0.0016 (16)	−0.0021 (17)
C8	0.033 (2)	0.050 (3)	0.034 (2)	0.004 (2)	0.0127 (19)	0.001 (2)
C9	0.056 (3)	0.055 (3)	0.033 (3)	0.004 (2)	0.018 (2)	−0.008 (2)
C10	0.032 (2)	0.056 (3)	0.026 (2)	0.001 (2)	−0.0023 (17)	−0.005 (2)
C11	0.023 (2)	0.065 (3)	0.045 (3)	0.000 (2)	0.0059 (19)	0.004 (2)
C12	0.042 (3)	0.044 (3)	0.069 (4)	−0.005 (2)	0.016 (3)	0.001 (3)
C13	0.040 (3)	0.041 (3)	0.042 (3)	−0.002 (2)	0.003 (2)	0.001 (2)
C14	0.055 (3)	0.042 (3)	0.056 (3)	−0.003 (2)	0.007 (2)	−0.001 (2)
C15	0.045 (3)	0.048 (3)	0.061 (3)	0.006 (2)	−0.002 (2)	−0.010 (3)
C16	0.047 (3)	0.037 (2)	0.039 (3)	−0.002 (2)	0.003 (2)	0.012 (2)
C17	0.061 (4)	0.070 (4)	0.060 (4)	−0.030 (3)	−0.023 (3)	0.017 (3)
C18	0.061 (4)	0.063 (3)	0.062 (4)	−0.003 (3)	0.009 (3)	0.038 (3)
Cl1	0.0365 (6)	0.0568 (7)	0.0392 (6)	0.0011 (5)	0.0027 (4)	−0.0050 (5)
Cl2	0.0330 (6)	0.0353 (5)	0.0324 (5)	−0.0002 (4)	0.0005 (4)	0.0006 (4)
Co1	0.0342 (3)	0.0336 (3)	0.0330 (3)	−0.0002 (2)	0.0007 (2)	0.0012 (2)
N1	0.0301 (19)	0.0342 (18)	0.0343 (19)	0.0050 (15)	0.0037 (15)	0.0049 (15)
N2	0.035 (2)	0.0317 (19)	0.046 (2)	0.0012 (15)	0.0081 (17)	0.0029 (16)
N3	0.034 (2)	0.0365 (18)	0.0241 (17)	−0.0024 (16)	0.0012 (14)	0.0022 (15)
N4	0.042 (2)	0.052 (2)	0.038 (2)	−0.0023 (19)	0.0052 (18)	−0.0134 (19)
N5	0.050 (3)	0.060 (3)	0.060 (3)	−0.011 (2)	0.024 (2)	−0.011 (2)
N6	0.047 (3)	0.045 (2)	0.052 (2)	0.006 (2)	−0.002 (2)	−0.014 (2)
N7	0.042 (2)	0.055 (2)	0.042 (2)	−0.012 (2)	−0.0004 (17)	0.0153 (19)
O1	0.0345 (17)	0.0401 (17)	0.0354 (17)	−0.0036 (14)	−0.0015 (13)	0.0082 (14)

### Geometric parameters (Å, °)

**Table:**

C1—N1	1.477 (5)	C10—H10A	0.9700
C1—C2	1.515 (6)	C10—H10B	0.9700
C1—H1A	0.9700	C11—C12	1.432 (8)
C1—H1B	0.9700	C11—H11A	0.9700
C2—N2	1.487 (6)	C11—H11B	0.9700
C2—H2A	0.9700	C12—N5	1.119 (6)
C2—H2B	0.9700	C13—N3	1.486 (6)
C3—N2	1.486 (6)	C13—C14	1.526 (7)
C3—C4	1.501 (7)	C13—H13A	0.9700
C3—H3A	0.9700	C13—H13B	0.9700
C3—H3B	0.9700	C14—C15	1.423 (7)
C4—N3	1.464 (6)	C14—H14A	0.9700
C4—H4A	0.9700	C14—H14B	0.9700
C4—H4B	0.9700	C15—N6	1.170 (6)
C5—N3	1.481 (5)	C16—O1	1.235 (5)
C5—C6	1.491 (6)	C16—N7	1.291 (6)
C5—H5A	0.9700	C16—H16	0.9300
C5—H5B	0.9700	C17—N7	1.405 (6)
C6—N1	1.477 (5)	C17—H17A	0.9600
C6—H6A	0.9700	C17—H17B	0.9600
C6—H6B	0.9700	C17—H17C	0.9600
C7—N1	1.478 (5)	C18—N7	1.426 (6)
C7—C8	1.531 (6)	C18—H18A	0.9600
C7—H7A	0.9700	C18—H18B	0.9600
C7—H7B	0.9700	C18—H18C	0.9600
C8—C9	1.458 (7)	Cl1—Co1	2.4382 (14)
C8—H8A	0.9700	Cl2—Co1	2.4096 (13)
C8—H8B	0.9700	Co1—O1	2.114 (3)
C9—N4	1.116 (6)	Co1—N2	2.194 (4)
C10—N2	1.470 (6)	Co1—N3	2.200 (4)
C10—C11	1.550 (6)	Co1—N1	2.232 (4)
			
N1—C1—C2	112.0 (3)	C14—C13—H13A	107.8
N1—C1—H1A	109.2	N3—C13—H13B	107.8
C2—C1—H1A	109.2	C14—C13—H13B	107.8
N1—C1—H1B	109.2	H13A—C13—H13B	107.1
C2—C1—H1B	109.2	C15—C14—C13	115.1 (5)
H1A—C1—H1B	107.9	C15—C14—H14A	108.5
N2—C2—C1	112.4 (4)	C13—C14—H14A	108.5
N2—C2—H2A	109.1	C15—C14—H14B	108.5
C1—C2—H2A	109.1	C13—C14—H14B	108.5
N2—C2—H2B	109.1	H14A—C14—H14B	107.5
C1—C2—H2B	109.1	N6—C15—C14	177.6 (6)
H2A—C2—H2B	107.9	O1—C16—N7	125.1 (5)
N2—C3—C4	111.7 (3)	O1—C16—H16	117.5
N2—C3—H3A	109.3	N7—C16—H16	117.5
C4—C3—H3A	109.3	N7—C17—H17A	109.5
N2—C3—H3B	109.3	N7—C17—H17B	109.5
C4—C3—H3B	109.3	H17A—C17—H17B	109.5
H3A—C3—H3B	107.9	N7—C17—H17C	109.5
N3—C4—C3	112.2 (4)	H17A—C17—H17C	109.5
N3—C4—H4A	109.2	H17B—C17—H17C	109.5
C3—C4—H4A	109.2	N7—C18—H18A	109.5
N3—C4—H4B	109.2	N7—C18—H18B	109.5
C3—C4—H4B	109.2	H18A—C18—H18B	109.5
H4A—C4—H4B	107.9	N7—C18—H18C	109.5
N3—C5—C6	112.8 (3)	H18A—C18—H18C	109.5
N3—C5—H5A	109.0	H18B—C18—H18C	109.5
C6—C5—H5A	109.0	O1—Co1—N2	87.78 (12)
N3—C5—H5B	109.0	O1—Co1—N3	168.01 (13)
C6—C5—H5B	109.0	N2—Co1—N3	80.93 (13)
H5A—C5—H5B	107.8	O1—Co1—N1	93.00 (13)
N1—C6—C5	112.5 (3)	N2—Co1—N1	81.07 (14)
N1—C6—H6A	109.1	N3—Co1—N1	81.30 (13)
C5—C6—H6A	109.1	O1—Co1—Cl2	90.45 (9)
N1—C6—H6B	109.1	N2—Co1—Cl2	173.80 (10)
C5—C6—H6B	109.1	N3—Co1—Cl2	100.34 (10)
H6A—C6—H6B	107.8	N1—Co1—Cl2	93.09 (10)
N1—C7—C8	117.6 (4)	O1—Co1—Cl1	91.12 (9)
N1—C7—H7A	107.9	N2—Co1—Cl1	93.38 (10)
C8—C7—H7A	107.9	N3—Co1—Cl1	93.58 (10)
N1—C7—H7B	107.9	N1—Co1—Cl1	172.94 (10)
C8—C7—H7B	107.9	Cl2—Co1—Cl1	92.60 (4)
H7A—C7—H7B	107.2	C6—N1—C1	113.8 (3)
C9—C8—C7	117.1 (4)	C6—N1—C7	111.0 (3)
C9—C8—H8A	108.0	C1—N1—C7	111.2 (3)
C7—C8—H8A	108.0	C6—N1—Co1	100.5 (2)
C9—C8—H8B	108.0	C1—N1—Co1	108.7 (3)
C7—C8—H8B	108.0	C7—N1—Co1	111.2 (2)
H8A—C8—H8B	107.3	C10—N2—C3	110.8 (3)
N4—C9—C8	177.2 (6)	C10—N2—C2	112.0 (3)
N2—C10—C11	115.5 (4)	C3—N2—C2	112.4 (3)
N2—C10—H10A	108.4	C10—N2—Co1	109.8 (3)
C11—C10—H10A	108.4	C3—N2—Co1	109.0 (3)
N2—C10—H10B	108.4	C2—N2—Co1	102.6 (3)
C11—C10—H10B	108.4	C4—N3—C5	113.5 (3)
H10A—C10—H10B	107.5	C4—N3—C13	112.0 (3)
C12—C11—C10	112.8 (4)	C5—N3—C13	112.0 (4)
C12—C11—H11A	109.0	C4—N3—Co1	102.5 (3)
C10—C11—H11A	109.0	C5—N3—Co1	108.2 (3)
C12—C11—H11B	109.0	C13—N3—Co1	108.0 (3)
C10—C11—H11B	109.0	C16—N7—C17	121.4 (4)
H11A—C11—H11B	107.8	C16—N7—C18	120.9 (5)
N5—C12—C11	178.4 (7)	C17—N7—C18	117.6 (4)
N3—C13—C14	118.2 (4)	C16—O1—Co1	119.1 (3)
N3—C13—H13A	107.8		

### Hydrogen-bond geometry (Å, °)

**Table:**

*D*—H···*A*	*D*—H	H···*A*	*D*···*A*	*D*—H···*A*
C1—H1A···Cl2^i^	0.97	2.75	3.658 (4)	157
C2—H2A···O1	0.97	2.58	3.120 (5)	116
C3—H3A···Cl2^i^	0.97	2.81	3.774 (4)	170
C7—H7A···Cl2	0.97	2.65	3.419 (4)	136
C10—H10A···O1	0.97	2.47	3.149 (6)	127
C10—H10B···Cl1	0.97	2.73	3.218 (4)	111
C11—H11A···Cl1^i^	0.97	2.62	3.525 (5)	156
C11—H11B···Cl2^ii^	0.97	2.65	3.502 (5)	147
C13—H13A···Cl1	0.97	2.72	3.460 (5)	133
C16—H16···Cl2	0.93	2.80	3.325 (5)	117
C17—H17A···O1	0.96	2.34	2.741 (7)	105

Symmetry codes: (i) *x*+1/2, −*y*+3/2, *z*+1/2; (ii) *x*+1, *y*, *z*.
